# Exome-based gene panel analysis in a cohort of acute juvenile ischemic stroke patients:relevance of *NOTCH3* and *GLA* variants

**DOI:** 10.1007/s00415-022-11401-7

**Published:** 2022-11-21

**Authors:** Johanna Härtl, Julia Hartberger, Silke Wunderlich, Isabell Cordts, Cemsel Bafligil, Marc Sturm, Regeneron Genetics Center, Dominik Westphal, Tobias Haack, Bernhard Hemmer, Benno David Ikenberg, Marcus Deschauer

**Affiliations:** 1 School of Medicine, Klinikum rechts der Isar, Department of Neurology, Technical University of Munich, Ismaningerstr. 22, 81675 Munich, Germany; 2 School of Medicine, Institute of Medical Genetics and Applied Genomics, Eberhard Karls University, Universitaetsklinikum Tuebingen, Tuebingen, Germany; 3 School of Medicine, Klinikum rechts der Isar, Department of Cardiology, Technical University of Munich, Munich, Germany; 4 School of Medicine, Klinikum rechts der Isar, Technical University of Munich, Institute of Human Genetics, Munich, Germany; 5 School of Medicine, Centre for Rare Diseases, Eberhard Karls University, Universitaetsklinikum Tuebingen, Tuebingen, Germany; 6 Regeneron Genetics Center, Tarrytown, NY USA; 7 Munich Cluster for Systems Neurology, (SyNergy), Munich, Germany

**Keywords:** Juvenile stroke, Ischemic stroke, Gene panel, Whole-exome sequencing, Stroke etiology

## Abstract

**Background:**

Genetic variants are considered to have a crucial impact on the occurrence of ischemic stroke. In clinical routine, the diagnostic value of next-generation sequencing (NGS) in the medical clarification of acute juvenile stroke has not been investigated so far.

**Material and methods:**

We analyzed an exome-based gene panel of 349 genes in 172 clinically well-characterized patients with magnetic resonance imaging (MRI)-proven, juvenile (age ≤ 55 years), ischemic stroke admitted to a single comprehensive stroke center.

**Results:**

Monogenetic diseases causing ischemic stroke were observed in five patients (2.9%): In three patients with lacunar stroke (1.7%), we identified pathogenic variants in *NOTCH3* causing cerebral autosomal-dominant arteriopathy with subcortical infarcts and leukoencephalopathy (CADASIL). Hence, CADASIL was identified at a frequency of 12.5% in the lacunar stroke subgroup. Further, in two male patients (1.2%) suffering from lacunar and cardioembolic stroke, pathogenic variants in *GLA* causing Fabry’s disease were present. Additionally, genetic variants in monogenetic diseases lacking impact on stroke occurrence, variants of unclear significance (VUS) in monogenetic diseases, and (cardiovascular-) risk genes in ischemic stroke were observed in a total of 15 patients (15.7%).

**Conclusion:**

Genetic screening for Fabry’s disease in cardioembolic and lacunar stroke as well as CADASIL in lacunar stroke might be beneficial in routine medical work-up of acute juvenile ischemic stroke.

**Supplementary Information:**

The online version contains supplementary material available at 10.1007/s00415-022-11401-7.

## Background

Genetic variants are considered to have a crucial impact on the occurrence of ischemic stroke. There are several well-defined monogenetic diseases causing ischemic stroke. Among these, cerebral small-vessel vasculopathies in lacunar stroke have been most frequently described. These include inter alia Fabry’s disease, cerebral autosomal-dominant arteriopathy with subcortical infarcts and leukoencephalopathy (CADASIL), cerebral autosomal-recessive arteriopathy with subcortical infarcts and leukoencephalopathy (CARASIL), and retinal vasculopathy with cerebral leukoencephalopathy (RCVL) [1]. Furtherly, pathogenic variants in genes associated with cardiomyopathy and arrhythmia in embolic stroke, metabolic disorders in large artery occlusion, and connective tissue diseases in dissections are considered relevant to stroke occurrence [1–7]. In addition to these monogenetic diseases, genome-wide association studies (GWAS) have identified several genes resulting in a significantly elevated risk for ischemic stroke [2, 8–11]. The incidence of genetic diseases associated with stroke is controversially discussed and is likely more frequent than previously estimated [7, 12]. So far, whole-exome sequencing (WES) in the medical clarification of juvenile ischemic stroke has been applied within two research studies only: In a cohort of 22 northern European patients with familial clustering stroke [3] and a large study of 10.000 ischemic stroke patients, which is currently performed in China but results are pending [13]. Exome-based gene panel analysis has mainly been performed in cerebral small vessel disease (CSVD) applying small gene panels only [7, 14]. A larger gene panel analysis consisting of 181 genes has so far only been performed in 53 pre-selected Chinese ischemic stroke patients [5]. In clinical practice, genetic analysis is to date not a part of the routine diagnostic workup in juvenile stroke patients admitted to stroke units. German guidelines on diagnostics for acute stroke clarification recommend genetic testing in selected patients based on clinical phenotyping only [15]. However, establishing a molecular diagnosis may have an impact on the diagnostic work-up, secondary prophylactic therapeutic strategies, coping strategies, and genetic counseling of families [16]. Given the broad availability of next-generation sequencing in clinical routine, either by WES or gene panels, we aimed to assess the added diagnostic value of a gene panel analysis as defined by the observed frequency of genetic causes of ischemic stroke in a large cohort of acute juvenile magnetic resonance imaging (MRI)- proven stroke patients admitted to our stroke unit for acute stroke work up.

## Material and methods

Written informed consent was obtained from all patients prior to WES and inclusion in our local biobank. The study was approved by the local ethics committee at the Technical University of Munich (project number 204/21S).

### Patient cohort

For patient identification, our local neurological biobank was applied. The neurological biobank of the Technical University of Munich is a registered biobank sampling patient biomaterial including DNA probes from patients with different neurological diseases [17]. In a retrospective approach, we identified all samples of included ischemic stroke patients treated from 2013 to 2018 at our comprehensive stroke care center. An ischemic stroke was diagnosed following clinical examination and confirmed by diffusion-weighted MRI sequences (DWI) in each case as part of standard care. We included patients in genetic analysis suffering from an ischemic stroke with a first stroke event up to and including 55 years of age, which will be characterized as juvenile stroke in the following [18]. Transient ischemic attacks (TIA) and patients with hemorrhagic stroke were not considered for analysis. No other in- or exclusion criteria were applied. Clinical baseline data were retrospectively retrieved from medical records.

### Standard treatment protocol

All included patients received standard stroke care, treatment and etiologic work-up according to national guidelines and following local standard operating procedures. This included Holter-ECG monitoring, sonographic examination of the blood supplying cervical and cranial vessels, cardiac echocardiography and laboratory examinations including lipid profile [19]. Stroke etiology was classified according to the international Trial of Org 10,172 in Acute Stroke Treatment criteria (TOAST) depending on diagnostic findings [20]. Family history was evaluated according to the letter of discharge and regarded as positive in cases of first-grade relatives suffering from ischemic stroke aged up to 55 years.

### Whole exome sequencing

WES was performed at the Regeneron Genetics Center (New York, USA) as previously described [21]. Generated sequences corresponding to a 20-fold coverage in > 80% of target bases. Sufficient quality was ensured by imposing quality control exclusions based on contamination score (contamination > 5% via verifyBamID software & heterozygous/homozygous ratio), gender concordance, sample duplication, and exome-genotype concordance.

### Definition of gene panel

Based on Ilinca et al*.*’s and Fang et al*.*’s suggestions for a gene panel on mendelian stroke, we defined a new panel consisting of 349 genes (Supplementary Table) [4, 5]. In 2020, Fang et al*.* proposed a panel consisting of 181 genes resulting in monogenetic diseases associated with stroke and 265 genes influencing the risk of stroke [5]. In 2018, Ilinca et al*.* described a panel of 120 genes with documented impact on stroke etiology in at least one patient, 62 genes with possible impact on stroke occurrence but lacking case report, and 32 risk genes detected by GWAS [4]. We combined both panels and added recent findings according to a search in PubMed. Consequently, the applied panel combined known monogenetic disorders causing ischemic stroke, genes resulting in a higher occurrence of cardiovascular risk factors, susceptibility genes for stroke and cardiovascular risk factors, and identified gene loci according to recent GWAS [2, 4, 5, 9, 11, 20].

### Genetic analysis

WES data analysis was performed using the megSAP pipeline (https://github.com/imgag/megSAP). For variant analysis, the GSvar graphical user interface was used (https://github.com/imgag/ngs-bits/tree/master/doc/GSvar) [22]. Applying the gene panel described above, variants were evaluated according to their impact, allelic frequency in control databases (gnomAD and 1000 Genomes projects), and presence in HGMD and ClinVar sources [23, 24]. Copy Number Variations and structure variants were assessed. The pathogenicity of the identified variants was determined according to the American College of Medical Genetics and Genomics (ACMG) guidelines [25]. Accordingly, we subsumed our findings into two subgroups: Pathogenic or likely pathogenic variants in monogenetic diseases causing ischemic stroke and additional findings in genetic analysis. Ethnicity of the patients was estimated by comparing the frequencies of uncorrelated single nucleotide polymorphisms (SNPs) of our patients with individuals of major continental ancestries (European, African, and East Asian) from the 1000 Genomes panel version 3.

### Statistical analysis

All statistical analyses were performed using SPSS (IBM, SPSS Statistics 28.0.1). Alpha error was set at 5%.

## Results

### Patient cohort

In total, 172 patients were identified with MRI-proven ischemic stroke aged up to 55 years. Baseline patient and stroke characteristics are depicted in Table 1. Most frequently observed were strokes of undetermined etiology (*n* = 77; 41.0%). A positive family history was documented in 9.9% (*n* = 17).

**Table 1:** Patient characteristics included into genetic analysis according to patient and stroke characteristics

Patient characteristics	
**Patient**	
Number of patients included	172
Age (years)	
* Median* * 1QT-3QT*	49 42–59
Gender	
* Female* * Male*	60/34.9% 112/65.1%
Ethnicity	
* European* *African* * East Asian*	169/98.3% 2/1.2% 1/0.6%
Arterial hypertension	55/32.0%
Diabetes mellitus	13/7.6%
Nicotine abuse	59/34.3%
Family history	17/9.9%
**Stroke**	
TOAST category	
* Large-artery atherosclerosis* * Cardioembolism* * Small-vessel occlusion* *Other determined etiology* * Undetermined etiology*	21/12.2% 11/6.4% 24/14.0% 39/22.7% 77/44.8%

*1QT*  first quartile, *3QT* third quartile, *Family History* regarded positive in patients with first-degree relatives suffering from ischemic stroke up to 55 years of age, *TOAST* Classification of Stroke etiology according to the international Trial of Org 10172 in Acute Stroke Treatment criteria

### Patients with pathogenic variants in monogenetic diseases causing ischemic stroke

In five European patients, we identified an underlying monogenetic disease causing an ischemic stroke. Patient characteristics are depicted in Table 2.

**Table 2:** Patients with pathogenic variants in monogenetic diseases causing ischemic stroke (CADASIL and Fabry’s disease)

Clinical and genetic findings in five patients with pathogenic variants in genes causing monogenetic diseases associated with ischemic stroke															
Clinical information							Genetic information								
No	Age	Sex	TOAST	Additional phenotypic features	FH	MRI characteristics	Gene	Inheritance	Disease	Genotype	c.DNA	AAC	Transcript	ACMG	MAF
1	44	f	3	Migraine	+	Extensive WMH without emphasize on temporopolar region Multiple lacunar defects Subcortical supra- and infratentorial and thalamic MB	*NOTCH3*	AD	CADASIL	Het	c.1672 > T	p.Arg558Cys	NM_000435.3	5	0.0001
2	54	f	3	Migraine	–	Confluent extensive WMH Supra- and infratentorial cortical, thalamic MB	*NOTCH3*	AD	CADASIL	Het	c.872G > A	p.Cys291Tyr	NM_000435.3	5	n/a
3	52	m	3	–	–	Confluent, temporal WMH No MB	*NOTCH3*	AD	CADASIL	Het	c.544C > T	p.Arg182Cys	NM_000435.3	5	0.0001
4	31	m	3	Angiokeratoma Hypohidrosis Hypoacusis Recurrent diarrhoea	+	Vertebro-basilar lacunar defects	*GLA*	X-linked recessive	Fabry’s disease	Hem	c.782dup	p.Trp262 LeufsTer3	NM_000169.3	5	n/a
5	44	m	2	Angiokeratoma Small fiber neuropathy Cardiomyopathy with atrial fibrillation	-	Embolic strokes in all territories	*GLA*	X-linked recessive	Fabry’s disease	Hem	c.547 + 1G > A		NM_000169.3	5	n/a

Genetic information on the gene, variant, genotype, amino acid change, transcript and MAF according to gnomAD are depicted. Clinical information contains patient characteristics (age, sex), stroke etiology, additional phenotype information, family history as well as magnetic resonance imaging characteristics found

*ACMG* American college of medical genetics, *ACMG 5* pathogenic, *ACMG 4* likely pathogenic, *AAC* Amino acid change, *AD* autosomal dominant, *Age* Age at first stroke event, *c.DNA* coding position, *FH* Family History, *Het.* heterozygous, *Hem.* hemizygous, *MAF* Minor allele frequency according to gnomAD, *MB* Micro bleeds, *MRI* Magnetic resonance imaging characteristics, *n/a* not available in gnomAD, *no.* number, *TOAST* Classification of Stroke etiology according to the international Trial of Org 10172 in Acute Stroke Treatment criteria, *TOAST 2* cardioembolism, *TOAST 3* small-vessel occlusion, *WMH* White matter hyperintensities

**Fig. 1:**
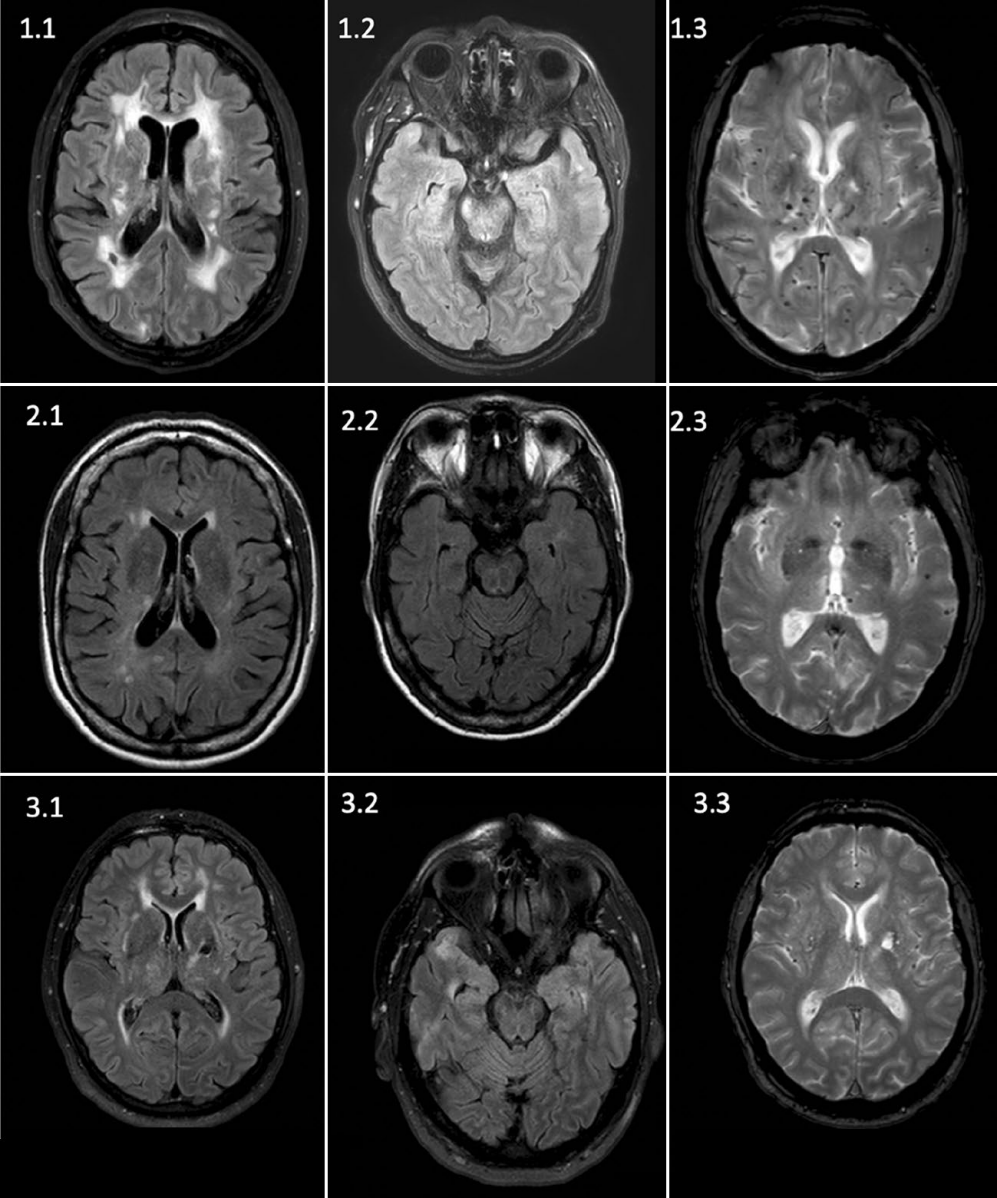
MRI images of the three CADASIL patients identified in our cohort. Patient 1 refers to the known CADASIL disease. 1.1: FLAIR sequence showing lacunar defects and extensive confluent white matter lesions, 1.2: FLAIR sequence, white matter lesions without emphasize on temporopolar region, 1.3: SWI sequence showing extensive microbleeds both thalamic and cortical. Patient 2 refers to the clinically suspected patient. 2.1: FLAIR sequence with extensive white matter lesions. 2.2: FLAIR Sequence, no emphasize on temporopolar, 2.3: T2* (“heme”) sequence showing supratentorial cortical microbleeds as well as thalamic microbleeds. Patient 3 refers to the clinically not suspected patient. 3.1 FLAIR sequence showing temporal white matter lesions and a lacunar defect in the thalamus, 3.2: FLAIR sequence, temporal white matter lesions, 3.3: T2* sequence, no microbleeds were shown

In three patients with lacunar stroke (patient number 1–3), we detected pathogenic, heterozygous missense variants in *NOTCH3* causing CADASIL (c.1672C > T, p.Arg558Cys; c.544C > T, p.Arg182Cys; c.872G > A, p.Cys291Tyr) [3, 26–28]. CADASIL had already been genetically proven in one case (patient 1) and was suspected in another case (patient 2) based on clinical and cerebral imaging characteristics. In patient 3, CADASIL was not suspected by the treating clinicians lacking both a positive family history and migraine. MRI characteristic showed periventricular white matter hyperintensities, which were solely attributed to the presence of cardiovascular risk factors prior to WES. MRI findings in FLAIR and T2* weighted sequence (“heme” sequence) of each patient are shown in Fig. 1.

In two further male patients, a hemizygous pathogenic variant in *GLA* was detected (c.782dup, p.Trp262LeufsTer3; c.547 + 1G > A). Fabry’s disease was known in both patients. One patient suffered from vertebrobasilar lacunar stroke (patient 4). The other one (patient 5) suffered from cardioembolic stroke, in association with cardiomyopathy and atrial fibrillation, being treated with insufficient oral anticoagulants.

### Patients with additional findings in genetic analysis

In total, we detected additional findings in 27 European patients. In one patient, two variants in cardiovascular risk factors became apparent. In contrast to the aforementioned patients, the detected variants were either pathogenic variants lacking association with stroke etiology, risk genes in ischemic stroke, or sufficient evidence for pathogenicity in stroke occurrence was missing*.* We consequently divided all observed variants into four subgroups:

(I) Likely pathogenic and pathogenic variants in genes resulting in monogenetic diseases that did not explain the occurrence of stroke (*n* = 2; 1.2%) (*TNNI3, KCQN1*),
(II) Variants of uncertain significance (VUS) that cannot conclude or exclude a monogenetic disease associated with stroke (*n* = 6; 3.5%) (*JAK2, COL4A1, COL5A1, NOTCH3*),
(III) Variants in genes associated with an increased risk of stroke (*n* = 5; 2.9%) (*RNF213, ABCC6, PROS1*), and
(IV) Variants in genes associated with cardiovascular risk factors (*n* = 15, 8.7%) (*ABCA1, APOB, LPL*).

The observed additional findings are listed in Table 3 depicting genetic and clinical information.

**Table 3:** Variants with uncertain impact on stroke are shown, which we characterized as additional findings.

	Clinical and genetic findings in 27 patients with variants in genes with possible impact on stroke but no evidence of a monogenetic stroke disease or with class 5 variants not explaining ischemic stroke												
	Clinical Information				Genetic Information								
	Age	TOAST	Sex	Additional phenotypic features	Gene	Inheritance	Phenotype generally associated	Genotype	c.DNA	AAC	Transcript	ACMG	MAF
***I***	53	5	m	Sclerosed aortic valve, No cardiomyopathy	*TNNI3*	AD	Hypertrophic Cardiomyopathy	Het	c.497C > T	p.Ser166Phe	NM_000363.5	5	0.0001
	33	5	f	Long-QT syndrome Follow-up event recorder: Sinus rhythm	*KCNQ1*	AD	Atrial Fibrillation, Long-QT	Het	c.1588C > T	p.Gln530Ter	NM_000218.2	5	0.0001
***II***	47	5	f	Hb: 11.2 g/dl Thrombocytes: 271 G/l	*JAK2*	Somatic, AD	Polycythemia vera	Het	c.3188G > A	p.Arg1063His	NM_00492.4	3	0.0047
	55	3	m	Hb: 15.1 g/dl Thrombocytes: 283 G/l		Somatic, AD	Polycythemia vera	Het	c.3188G > A	p.Arg1063His	NM_00492.4	3	0.0047
	53	1	m	Hb: 16.9 g/dl Thrombocytes: 288 G/l		Somatic, AD	Polycythemia vera	Het	c.3188G > A	p.Arg1063His	NM_00492.4	3	0.0047
	34	5	*m*		*COL4A1*	AD	Cerebral Small Vessel Disease	Het	c.4970C > T	p.Thr1657Met	NM_01845.6	3	0.0001
	54	3	*m*		*NOTCH3*	AD	CADASIL	Het	c.5129G > A	p.Gly1710Asp	NM_000435.3	3	0.0005
	51	4	m	ICA Dissection	*COL5A1*	AD	Ehlers-Danlos, classic type, Fibromuscular Dysplasia	Het	c.4307C > T	p.Pro1436Leu	NM_000093.5	3	0.0001
***III***	55	1	m	High-grade stenosis of the ICA left. Moderate stenosis of contralateral ICA *CVRF: moderate AHT*	*RNF213*	Susceptibility gene	Moyamoya Disease	Het	c.12055C > T	p.Arg4019Cys	NM_001256071.3	2/3	0.0010
	42	5	*m*		*ABCC6*	AR	Pseudoxanthoma elasticum	Het	c.3421C > T	p.Arg1141Ter	NM_001171.6	5	0.0014
	55	2	f			AR	Pseudoxanthoma elasticum	Het	c.1171A > G	p.Arg391Gly	NM_001171.6	5	0.0056
	44	5	m			AR	Pseudoxanthoma elasticum	Het	c.1232A > G	p.Asn411Ser	NM_001171.6	5	0.0001
	37	5	f	Strokes in all territories, PFO shown by TEE *Prot. S activity not measured*	*PROS1*	AD/AR	Thrombophilia due to Protein S deficiency	Het	c.233C > T	p.Thr78Met	NM_000313.4	4	0.0002
***IV***	47	5	m	HDL: 41 mg/dl	*ABCA1*	AR	Tangier Disease	Het	c.5398A > C	p.Asn1800His	NM_005502.4	5	0.0007
	46	5	f	HDL: 48 mg/dl		AR	Tangier Disease	Het	c.5398A > C	p.Asn1800His	NM_005502.4	5	0.0007
	54	5	f	HDL: 33 mg/dl (–)		AR	Tangier Disease	Het	c.5398A > C	p.Asn1800His	NM_005502.4	5	0.0007
	52	1	m	HDL 32 mg/dl (–)		AR	Tangier Disease	Het	c.1196 T > C	p.Val399Ala	NM_005502.4	4/5	0.0037
	44*	4	m	Moyamoya Disease Cholesterol 174 mg/dl HDL 25 mg/dl (–)		AR	Tangier Disease	Het	c.6083C > T	p.Ala2028Val	NM_005502.4	3	0.0002
	*				*APOB*	AD	Hypercholesterolemia, familial	Het	c.13288 T > A	p.Ser4430Thr	NM_000384.3	3	0.0001
	41	5	m	Cholesterol 221 mg/dl (+)		AD	Hypercholesterolemia, familial	Het	c.5269C > G	p.Leu175Val	NM_000384.3	3	0.0001
	53	3	m	Cholesterol 228 mg/dl (+)		AD	Hypercholesterolemia, familial	Het	c.689G > C	p.Gly230Ala	NM_000384.3	3	0.0001
	55	1	m	Cholesterol 228 mg/dl (+)		AD	Hypercholesterolemia, familial	Het	c.11401 T > A	p.Ser3801Thr	NM_000384.3	3	0.0012
	35	1	m	Cholesterol 166 mg/dl, Triglycerides 314 mg/dl (+), HDL 18 mg/dl (–)	*LPL*	AD	Combined Hyperlipidemia, familial	Het	c.953A > G	p.Asn318Ser	NM_000237.3	3	0.0145
	50	1	f	Cholesterol 361 mg/dl (+), Triglycerides 780 mg/dl (+), LDL 127 mg/dl (+), HDL 42 mg/dl		AD	Combined Hyperlipidemia, familial	Het	c.953A > G	p.Asn318Ser	NM_000237.3	3	0.0145
	55	5	m	Cholesterol 213 mg/dl (+), Triglycerides 261 mg/dl (+), LDL 150 mg/dl (+), HDL 30 mg/dl (–)		AD	Combined Hyperlipidemia, familial	Het	c.953A > G	p.Asn318Ser	NM_000237.3	3	0.0145
	48	5	f	Cholesterol 209 mg/dl (+), Triglycerides 164 mg/dl, LDL 164 mg/dl (+), HDL 30 mg/dl (–)		AD	Combined Hyperlipidemia, familial	Het	c.953A > G	p.Asn318Ser	NM_000237.3	3	0.0145
	52	5	m	Cholesterol 258 mg/dl (+), Triglycerides 287 mg/dl (+), LDL 188 mg/dl (+), HDL 40 mg/dl		AD	Combined Hyperlipidemia, familial	Het	c.953A > G	p.Asn318Ser	NM_000237.3	3	0.0145
	51	1	m	Cholesterol 191 mg/dl, Triglycerides 200 mg/dl, LDL 145 mg/dl (+), HDL 33 mg/dl (–)		AD	Combined Hyperlipidemia, familial	Het	c.286G > A	p.Val96Leu	NM_000237.3	3/4	0.0001

Genetic information on the variant detected as well as clinical information on the patient are depicted. Additional findings were divided into four subgroup as depicted in the results

*ACMG* American college of medical genetics, *ACMG 5* pathogenic, *ACMG 4* likely pathogenic, *ACMG 3* variant of unknown significance, *ACMG 2* likely benign, *AAC* Amino acid change, *AD* autosomal dominant, *Age* Age at first stroke event, *AHT* arterial hypertension, *c.DNA* coding position, *Cholesterol* target value < 190 mg/dl, *FH* Family History, *Hb* Hemoglobin standard value depending on sex from 12 to 18 g/dl, *HDL* High Density Lipoprotein Cholesterol with a standard value 35–65 mg/dl, *Het.* heterozygous, *Hom.* homozygous, *ICA* Internal Carotid Artery, *LDL* Low Density Lipoprotein Cholesterol with a target value < 100 mg/dl, *MAF* Minor allele frequency according to gnomAD, *MB* Micro bleeds, *MRI* Magnetic resonance imaging characteristics, *n/a* not available in gnomAD, *TEE* transesophageal echocardiography, *TOAST* Classification of Stroke etiology according to the international Trial of Org 10172 in Acute Stroke Treatment criteria, *TOAST 1* large-artery atherosclerosis, *TOAST 2* cardioembolism, *TOAST 3* small-vessel occlusion, *TOAST 4* stroke of other determined etiology, *TOAST 5* stroke of undetermined etiology, *Triglycerides* target value < 200 mg/dl, *Thrombocytes G/l* 10^9^/l standard value 150–450 G/l, *WMH* White matter hyperintensities, * = same patient with multiple variants, (+) = elevated compared to target value, (–) lowered compared to target value.

## Discussion

We present an exome-based large gene panel analysis of patients with MRI-proven acute juvenile ischemic stroke representing a real-world study population admitted to a single comprehensive stroke care center. As a major finding, our data shows the clinically relevant frequency of CADASIL and Fabry’s disease in juvenile ischemic stroke patients.

With a frequency of 12.5% in lacunar stroke, the occurrence of CADASIL in our cohort is more prevalent than described in prior studies: Tan et al*.* detected CADASIL in eleven patients in their selected patient cohort of 950 lacunar stroke patients ≤ 70 years of age resulting in a total frequency of 1.2% [7]. Compared to our data, the difference in the observed frequency could be attributable to the varying age inclusion criteria. In another study, Ilinca et al*.* detected one pathogenic *NOTCH3* variant in their cohort of 22 juvenile stroke patients ≤ 56 years with familial clustering of ischemic stroke corresponding to a frequency of 4.5% [3]. In contrast to these highly preselected studies, our data show that CADASIL needs to be considered in a comparatively unselected cohort and everyday juvenile stroke care.

Furtherly, three considerations for the indication of genetic testing on *NOTCH3* were apparent. Firstly, given the case of CADASIL with negative family history in our patient cohort and as de-novo mutations in CADASIL have been reported before, genetic testing on *NOTCH3* solely based on family history may be insufficient [29]. Secondly, imaging findings varied regarding localization of white matter lesions as well as the occurrence of microbleeds and, as shown in one case, combined with the presence of cardiovascular risk factors may bias clinical judgement [6]. Additionally, ethnicity has been shown to have an impact on the clinical as well as the radiological phenotype of CADASIL: The Asian population was found to present less migraine and seizures, but more intracerebral hemorrhage and a difference in the localization of white matter hyperintensities [30, 31]. Our data hence encourages genetic testing on *NOTCH3* in all patients suffering from juvenile lacunar stroke.

Regarding Fabry’s disease, the frequency of 1.2% observed in our patient cohort confirms previous findings: A meta-analysis including nine studies by Shi et al*.* showed a prevalence of 0.4–2.6% of Fabry’s disease in juvenile stroke [32]. Typically, as observed in one patient with the p.Trp262LeufsTer3 variant, patients with Fabry’s disease present microvascular changes on MRI scans [33]. Cardioembolic stroke has additionally been reported in Fabry’s disease [33–35]. Our data underline the importance of screening for Fabry’s disease in lacunar juvenile stroke as well as cardioembolic and stroke of undetermined etiology.

We did not detect pathogenic variants in other genes associated with monogenetic diseases causing stroke. This is in accordance with previously published studies: In 950 patients Tan et al*.* detected only three cases with pathogenic variants in other monogenetic diseases causing stroke (*HTRA1*, *COL4A1)* apart from *NOTCH3* [7]. Similarly, Coste et al*.* showed that *COL4A1* and *COL4A2* as well as *APP, TREX1* and *HTRA1* were much less frequent than pathogenic variants in *NOTCH3* in patients suffering from cerebral small vessel disease (CSVD) advised on genetic testing [14].

In addition to the described pathogenic variants, we detected heterozygous VUS in four genes associated with monogenetic diseases causing an ischemic stroke (Table 3/II). A novel *JAK2* variant (p.Arg1063His) was described in a patient with embolic stroke of undetermined etiology (ESUS) and familial clustering of ischemic juvenile stroke [3]. As prothrombotic status with and without erythrocytosis in patients carrying *JAK2* variants has been shown, this variant might be considered relevant in ischemic stroke etiology [3]. In our patient cohort, the variant could be detected in a total of three patients (1.7%), which is more frequent than listed in controls (0.4–0.7%, Table 2). However, the phenotype of patients in our cohort was diverse and only one patient suffered from ESUS. Concluding, the impact of this variant on stroke etiology requires future characterization. Further, in one patient with a dissection of the internal carotid artery, we detected a VUS in *COL5A1* (p.Pro1436Leu), a gene associated with fibromuscular dysplasia and artery dissection. In a retrospective approach, the impact of the variant on stroke etiology cannot be proven. Furtherly, we detected a VUS in *COL4A1* (p.Thr1657Met) previously described as a novel variant in CSVD [7]. However, the stroke phenotype did not match. Lastly, a VUS in *NOTCH3 (*p.Gly1710Asp) was found in a patient with lacunar stroke, which has been reported in association with cerebral white matter lesions but not ischemic stroke [36, 37]. Considering the lack of a pathognomonic cysteine change and the conflicting interpretation of prediction tools (SIFT, Polyphen2), the variant seemed less likely relevant in stroke etiology.

Incidental findings in monogenetic analysis became apparent in two patients (1.2%) (Table 3/I): In one possibly pre-symptomatic patient, we detected a pathogenic variant in *TNNI3* (p.Ser166Phe) causing autosomal dominant hypertrophic cardiomyopathy [38]. In another patient with Long-QT syndrome, we detected a pathogenic loss-of-function variant in *KCNQ1* (p.Gln530Ter) [39]. In follow-up event recorder documentation, atrial fibrillation was not detected, a phenotype associated with gain-of-function variants in this gene. Compared to literature, the observed frequency of incidental findings in genetic testing can be expected [40]. Nevertheless, it addresses the ethical responsibility in genetic counselling and stresses the medical and possibly long-term impact of an incidental genetic finding on the patient and their family.

Risk genes for ischemic stroke became apparent in five patients (Table 3/III). In three patients, we detected pathogenic variants in *ABCC6* (p.Arg1141*, p.Arg391Gly, p.Asn411Ser) [10, 41]. Bi-allelic variants are known to cause autosomal recessive pseudoxanthoma elasticum [42], a connective tissue disease resulting inter alia in arterial calcification with a high incidence of cardiovascular events including cerebral ischemia. In patients with heterozygous variants in this gene, an elevated odds ratio (OR) of 4.9 for ischemic stroke has been shown [10]. Secondly, in a patient with patent foramen ovale (PFO) and clinically assumed paradox embolic stroke, we detected a likely pathogenic variant in *PROS1,* which is in the heterozygous state associated with thrombophilia due to protein S deficiency (p.Thr78Met) [43]. Due to the retrospective analysis, protein S activity was not measured in our patient and causative connection cannot be proven. Lastly, in one patient, a heterozygous variant (p.Arg4019Cys) in *RNF213*, a susceptibility gene in Moyamoya disease, was present [44, 45]. However, the patient did not present angiographic signs of Moyamoya disease and incomplete penetrance has been shown before [45].

Summarizing, the frequent finding of additional genetic variants in 15.7% of our patient cohort emphasizes the possibility that rarer genetic diseases may account for juvenile ischemic stroke and highlights the relevance of future genetic research in stroke etiology. It has been shown before, that a genomic risk score may outperform classic risk factors concerning the predictive values of stroke recurrence [46]. The broad spectrum of genetic findings furtherly represents the diverse etiologies of ischemic stroke and stresses the necessity of individual stroke care and follow-up.

The strength of our study is a real-world study population. This implies that results may be applicable to other stroke centers. Limitations of this study refer to its retrospective approach. This implies that the genetic data of family members were not available for analysis. Further, the study was limited due to the small size of the study population and etiologic subgroups. The monocentric design and the dependency on our local biobank cannot exclude a possible selection bias. Lastly, the impossibility to detect intronic variants by WES is of importance.

To our knowledge, there are currently no recommendations on standardized genetic testing in stroke or even juvenile stroke patients [15]. Our data showed that CADASIL and Fabry’s disease constitute a frequent etiology of acute juvenile ischemic stroke and should hence already be considered in the initial work-up of patients. Given the divers stroke etiologies observed, screening for Fabry’s disease should be considered in all patients suffering from juvenile stroke of cardioembolic, lacunar, and undetermined etiology. Additionally, we suggest routine genetic testing for CADASIL in all patients suffering from a juvenile lacunar stroke. As described above, the presence of common cardiovascular risk factors may conceal the underlying genetic disease, consequently deteriorate the medical clarification of lacunar strokes, and interfere with the definition of clinically applicable red flags [6]. Only in cases with a more refined phenotype indicating a monogenetic stroke etiology based on characteristic extra- and intracerebral features or conclusive family history, a gene panel analysis based on NGS may have an additional diagnostic benefit. In those patients, exome and genome sequencing has two advantages over panel-based approaches targeting a predefined set of genes: Apart from the methodological advantages in detecting certain types of genetic variations, there is the prospect of identifying novel disease genes.

## Supplementary Information

Below is the link to the electronic supplementary material.

Supplementary file1 (XLSX 70 KB)

Supplementary file2 (DOCX 24 KB)
